# Isotope Label-Aided Mass Spectrometry Reveals the Influence of Environmental Factors on Metabolism in Single Eggs of Fruit Fly

**DOI:** 10.1371/journal.pone.0050258

**Published:** 2012-11-21

**Authors:** Te-Wei Tseng, June-Tai Wu, Yu-Chie Chen, Pawel L. Urban

**Affiliations:** 1 Department of Applied Chemistry, National Chiao Tung University, Hsinchu, Taiwan; 2 Institute of Molecular Medicine, College of Medicine, National Taiwan University, Taipei, Taiwan; 3 Department of Medical Research, National Taiwan University Hospital, Taipei, Taiwan; 4 Research Center for Developmental Biology and Regenerative Medicine, National Taiwan University, Taipei, Taiwan; 5 Institute of Molecular Science, National Chiao Tung University, Hsinchu, Taiwan; Yale School of Medicine, United States of America

## Abstract

In order to investigate the influence of light/dark cycle on the biosynthesis of metabolites during oogenesis, here we demonstrate a simple experimental protocol which combines *in-vivo* isotopic labeling of primary metabolites with mass spectrometric analysis of single eggs of fruit fly (*Drosophila melanogaster*). First, fruit flies were adapted to light/dark cycle using artificial white light. Second, female flies were incubated with an isotopically labeled sugar (^13^C_6_-glucose) for 12 h – either during the circadian day or the circadian night, at light or at dark. Third, eggs were obtained from the incubated female flies, and analyzed individually by matrix-assisted laser desorption/ionization (MALDI) mass spectrometry (MS): this yielded information about the extent of labeling with carbon-13. Since the incorporation of carbon-13 to uridine diphosphate glucose (UDP-glucose) in fruit fly eggs is very fast, the labeling of this metabolite was used as an indicator of the biosynthesis of metabolites flies/eggs during 12-h periods, which correspond to circadian day or circadian night. The results reveal that once the flies adapted to the 12-h-light/12-h-dark cycle, the incorporation of carbon-13 to UDP-glucose present in fruit fly eggs was not markedly altered by an acute perturbation to this cycle. This effect may be due to a relationship between biosynthesis of primary metabolites in developing eggs and an alteration to the intake of the labeled substrate – possibly related to the change of the feeding habit. Overall, the study shows the possibility of using MALDI-MS in conjunction with isotopic labeling of small metazoans to unravel the influence of environmental cues on primary metabolism.

## Introduction

Circadian clock helps biological organisms to control their physiological and developmental processes [1]. Biological rhythmicity is common in nature, with environmental factors – such as light and temperature – synchronizing internal time of the organisms to the 24-h cycle [2]. Disruption of circadian rhythms can also lead to metabolic disorders [3]. Thus, studying relationships between circadian rhythms and metabolism may contribute to a better understanding of the mechanism and robustness of the biological clock. Most concepts in molecular regulation of circadian rhythms in eukaryotic cells have been based on transcription-translation feedback loops; however, a recent study demonstrated the existence of a phenotypic circadian clock in red blood cells – *i.e.* in a biological system where no transcription occurs [4]. Small metazoa are convenient models for studying circadian rhythms; one of them is fruit fly (*Drosophila melanogaster*) [5]. In fact, fruit fly is one of the most studied invertebrates, and a large amount of scientific information about this species is currently available [6], [7]. Although fruit fly has served as a model organism in several studies of circadian rhythms [8]–[10], limited data is currently available on the influence of adaptation of fruit fly to light/dark cycle on primary metabolism in this species. Feeding and metabolic activities of fruit fly are affected by the existence of circadian rhythms [11]. However, the small volumes of samples obtained from individual flies disable the possibility of analyzing metabolites using conventional analytical tools, and following the effects of light/dark cycle on biosynthesis of metabolites in single fly organs.

**Figure 1:**
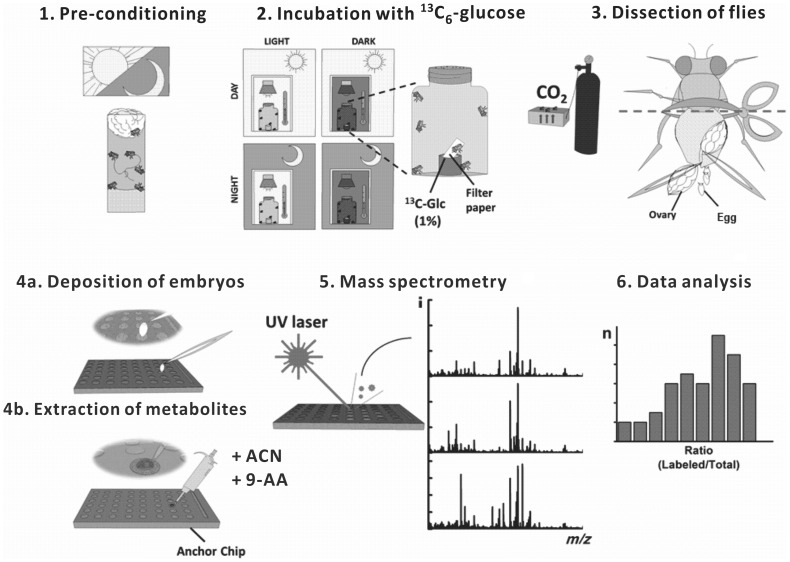
Experimental design and chemical analysis workflow. (1) pre-conditioning (entrainment) of the culture stock (adaptation to the 12/12-h (light/dark) cycle); (2) incubation of female fruit flies with ^13^C_6_-glucose solution; (3) dissection of the anesthetized flies; (4a/4b) preparation of individual eggs for mass spectrometric analysis; (5) mass spectrometry, and (6) data analysis.

There exist a number of analytical techniques applicable to the analysis of metabolites in biological samples; two prominent examples include the coupling of liquid chromatography (LC), or gas chromatography (GC) with mass spectrometry (MS). The GC-MS platform enables analysis of volatile analytes in large numbers of samples; it offers high sensitivity, reproducibility, and can easily be automated [12]. Implementation of chromatographic techniques usually necessitates pre-treatment of the samples. In most cases, this renders LC-MS and GC-MS inadequate to analysis of samples smaller than ∼1 mm. In the past few years, matrix-assisted laser desorption/ionization (MALDI) [13] mass spectrometry has emerged as an enabling tool for the metabolic profiling of microscale samples [14], [15]; it provides what the other analytical tools cannot offer: compatibility with micrometer-scale samples (single cells, egg chambers, *etc.*), high sensitivity, and it does not require complicated sample preparation. MALDI-MS takes advantage of laser beam to volatilize and ionize compounds embedded in a chemical matrix [16]. The matrix molecules absorb energy from the laser light, and transfer it onto the analyte molecules, which become ionized in the gas phase. A mass spectrum can be obtained right after a few shots of laser light impinge on a sample/matrix deposit. A major disadvantage of MALDI-MS is its limited quantitative capability – a characteristic attributed to the limitations of sample preparation protocols, and ion suppression effects. When using MALDI-MS in the studies of metabolism, one possible solution to this problem is the implementation of stable-isotope labels [17], [18]. The *in-vivo* labeling of fruit flies with stable isotopes – in combination with LC-MS – has already gained an insight into the proteome of fruit fly [19]. Therefore, to mitigate the problems related to the signal variability in MALDI-MS, here we have also implemented *in-vivo* isotopic labeling of metabolites in fruit flies.

In order to investigate the influence of circadian adaptation on egg metabolism, we have implemented the following protocol (**Figure 1**): First, fruit flies are adapted to light/dark cycle using artificial white light. Second, female flies are incubated with ^13^C-labeled glucose (the only carbon source) for 12 h – either during circadian day or circadian night, either at light or at dark. The labeled carbohydrate is ingested by the flies, and metabolized. Third, samples of unfertilized eggs are obtained from the incubated flies, and the relative abundances of metabolite isotopologues present in individual eggs are determined by MALDI-MS. We sought answers to the following questions: (i) Will ^13^C-labeled glucose be used as a carbon source in primary metabolism, and will the ^13^C atoms be incorporated into metabolites in individual eggs? (ii) Can MALDI-MS provide useful quasi-quantitative results (*i.e.* without performing absolute quantification), which would reflect the treatment applied to the fly stocks (*e.g.* variation of temperature or light)? (iii) Does the light/dark cycle affect metabolite labeling in female fruit flies?

We have opted for measuring metabolite levels in the samples composed of single eggs. This choice was made due to several reasons: (i) Eggs can be considered as a sink for the absorbed nutrients and primary metabolites. (ii) Eggs occupy substantial volume in the fly abdomen, and the amount of the biological material contained in single egg is sufficient for the analysis by MALDI-MS. (iii) It is relatively easy to obtain multiple eggs from individual flies through manual dissection. (iv) Eggs are more compact, less vulnerable to osmolarity changes and mechanical stress, as compared with other fruit fly organs (*e.g.* ovarioles, gut, brain) which can be sampled for chemical analysis. Fruit fly eggs measure approximately half millimeter, which can be regarded as an adequate size of a sample to be analyzed by MALDI-MS following careful sample preparation.

## Materials and Methods

### Fly Stocks

Fruit flies (*Drosophila melanogaster; w^1118^*– a normal control strain purchased from the *Drosophila* Stock Center in the Department of Biology at Indiana University, USA) were reared on a standard medium (water, yeast, soy flour, yellow cornmeal, agar, light corn syrup, propionic acid) loaded into plastic vials. Typically, the stock culture was maintained at room temperature. The default photoperiod was 16-hr day/8-hr night; however, during the entrainment period (before the experiments related to the circadian rhythms), it was changed to 12-hr day/12-hr night.

### Isotopic Labeling

Female and male fruit flies (typical age: ∼ 1 week) were separated under stereomicroscope (Zeiss, Munich, Germany), and the female individuals were subsequently transferred into 100-mL glass vials (Richiden-Rika Glass Company, Kobe City, Japan). A plastic cap with a stripe of filter paper wetted with 1% ^13^C_6_-glucose solution in water was inserted to each of the vials, so that the flies were exposed to the ^13^C_6_-labeled glucose during the following hours/days. In most experiments, the vials were put inside an incubator in order to control the temperature. Most flies survived at least 7 days under these conditions. During the labeling experiments, illumination was provided by a light-emitting diode (LED) lamp (white light; Aliiv, Taipei, Taiwan), which ensured the illuminance of ∼4000 lux. During the entrainment period, weaker light (∼150 lux) was used.

**Figure 2:**
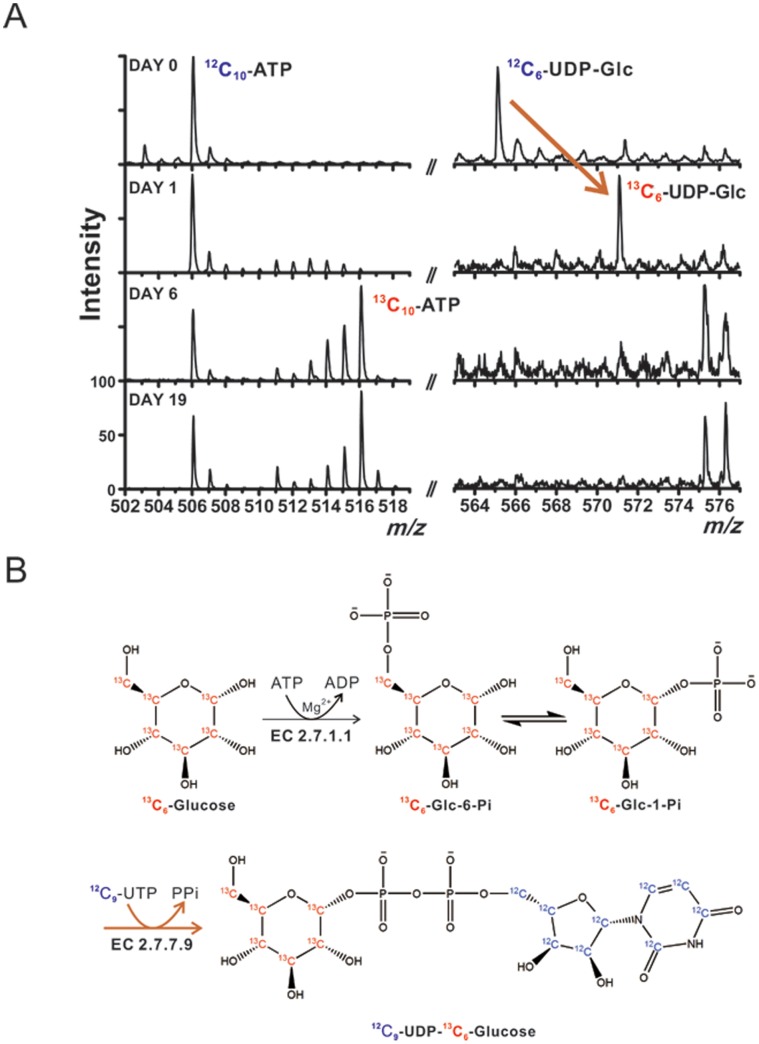
Isotopic labeling of ATP and UDP-glucose in single eggs. (A) MALDI mass spectra obtained from fruit flies following 1, 6, and 19 days of incubation with the ^13^C_6_-glucose solution. (B) Reaction scheme of labeling glucose moiety in UDP-glucose with ^13^C_6_-glucose.

### Dissection of Flies and Sample Preparation

Before the dissection, flies were anesthetized with carbon dioxide gas. Heads and abdomens were separated from thoraxes using miniature scissors (Vannas-Tübingen Spring; FST, Foster City, California). A set of precise tweezers (Dumont, Munich, Germany) was used to remove ovaries, and obtain unfertilized eggs (for a reference to the dissection protocol, see, for example: ref. [20]). After a brief washing in phosphate buffered saline solution, the eggs were transferred – one-by-one – onto separate recipient sites (i.d. 0.4 µm) of a metal AnchorChip plate (Bruker Daltonics, Bremen, Germany). Following the deposition of the eggs onto the target plate, an aliquot of 0.5 µL 1∶1 (v/v) acetonitrile/water solution (after initial optimization) was pipetted to initiate extraction of metabolites from the egg; then, a 0.5-µL aliquot of 6 mg mL^−1^ 9-aminoacridine (Sigma-Aldrich, St Louis, USA; MALDI matrix) solution in acetone (Merck, Darmstadt, Germany) was added. Following the evaporation of the solvents, the resulting sample/matrix deposits were ready for analysis by mass spectrometry. Note that 9-aminoacridine is regarded as a carcinogen, and personal safety equipment must be used when handling preparations of this compound. Animal tissue residues are disposed off as biological waste.

### Mass Spectrometry

During the mass spectrometric analysis, we used the AutoFlex III MALDI-time-of-flight (TOF)-MS from Bruker Daltonics, which is equipped with a solid-state laser (λ = 355 nm). Negative-ion mode was used with the following settings: ion source 1, −19.0 kV; ion source 2, −16.6 kV; lens, −8.45 kV; delay time, 0 ns. The Smartbeam-laser focus was set to “small” (∼40 µm); 100 shots were fired at each sample spot with the preset frequency of 50 Hz. The mass range was set to 400–1000 Da, and the suppression threshold was set to 400 Da, so that the low-*m/z* ions (including the matrix ions) could not reach the detector.

### Data Treatment

The MS data were acquired using the FlexControl software (version 3.0; Bruker Daltonics), and further analyzed with the FlexAnalysis software (version 3.0; Bruker Daltonics). The output data were further used to calculate the ratios of peak areas at the *m/z*: 571/(565+571). These ratio values were used to plot histograms with the bin width of 0.1. Statistical analysis (one- and two-sample Kolmogorov-Smirnov test, Wilcoxon rank sum test) was conducted using the MATLAB software (version 7.6.0.324 (R2008a), MathWorks, Natick, USA). Curve fitting was conducted using the SPSS software (version 19, IBM Corp., New York, USA). Other data were treated and displayed using the Origin Pro software (version 8; Origin Lab Corporation, Northampton, USA).

**Figure 3:**
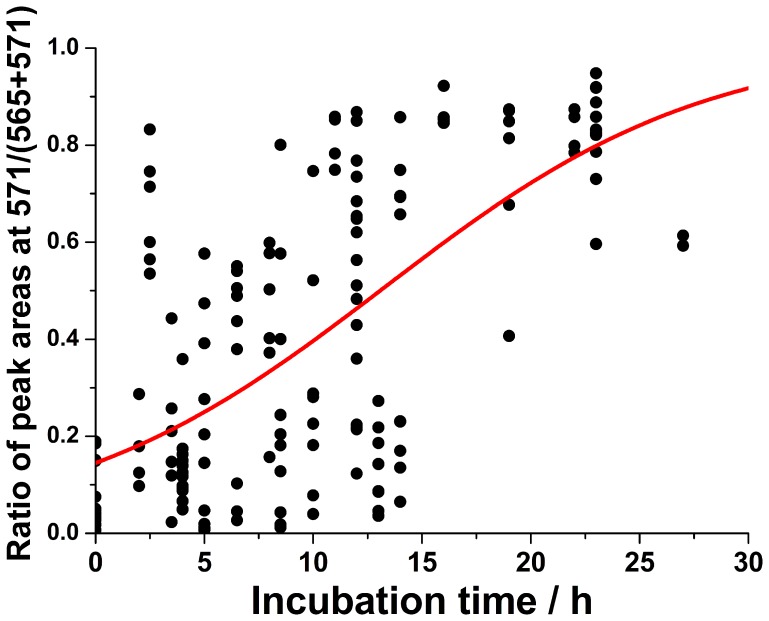
Progressive incorporation of the ^13^C-label to UDP-glucose in fruit fly eggs. The data points were fitted with a sigmoid function: *y* = 1.013·(1/(1+*e* ^(−0.135*x*+1.792)^)). During this experiment, the flies were incubated with ^13^C_6_-glucose and illuminated with white light (∼ 4000 lux). Note that the sampling intervals (as projected onto the *x*-axis) are coincidentally not constant.

## Results and Discussion

### Preliminary Experiments and Optimization

Implementation of the proposed analytical workflow (**Figure 1**) has been preceded by a series of preliminary experiments. Initially, the *in-situ* extraction of metabolites from samples was tested and optimized. Metabolites were passively extracted from eggs on the MALDI target (AnchorChip, Bruker Daltonics), and co-crystallized with the matrix compound (9-aminoacridine). We chose to use acetonitrile as the extraction solvent since it has widely been used for extraction of cellular metabolites (*e.g.* ref. [21]). During the optimization step, we tested mixtures of acetonitrile with water at different volume ratios. 9-Aminoacridine is a suitable matrix for MALDI-MS analysis of metabolites in the negative-ion mode [22]. Signals corresponding to several primary metabolites – which leaked out of the egg – could readily be identified in the MALDI mass spectra (*e.g.* **Figure S1**).

**Figure 4:**
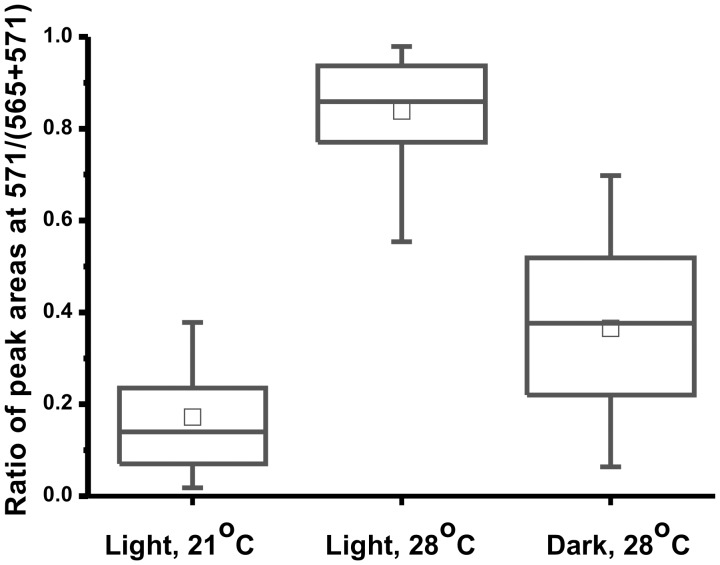
Influence of temperature (21 *vs.* 28°C) and illumination (dark *vs.* light) on the labeling of glucose moiety in UDP-glucose molecules extracted from individual eggs. Female flies were incubated with ^13^C_6_-glucose solution during 24 h. The default conditions were: white light on, ∼4000 lux (in the study involving the change of temperature); temperature, 28°C (in the study involving the change of illumination).

The optimization of the sample preparation involved experiments in which the percentage of acetonitrile in the extraction solution as well as the concentration of 9-aminoacridine in the matrix cocktail were varied. Based on the measurements of signal-to-noise (*S/N*) ratios in the resulting spectra (**Figures S2** and **S3**), we chose acetonitrile mixed with water at the ratio 1∶1 (v/v) as the extraction solvent, and 6 mg mL^−1^ 9-aminoacridine solution in acetone as the MALDI matrix cocktail. It is believed that at a low percentage of acetonitrile, biological membranes are not destabilized/degraded sufficiently to support leakage of the contents of the cells. On the other hand, at high percentage of acetonitrile, evaporation of the extraction solution is too fast, and – as a result – the extraction time is too short, and the amounts of extracted metabolites are insufficient to produce intense MS signals. It should also be pointed out that – unlike the common extraction protocols used in metabolite analysis – the on-target extraction is almost completely passive, *i.e.* without prior sample degradation, grinding, shaking, or stirring. Metabolites need to be extracted despite the presence of the outer chorion layer protecting the egg. Although many standard protocols include the removal of the outer layer, the *in-situ* extraction process was conducted without prior dechorionation of the eggs. Based on the preliminary experiments, the outer protective layer of the eggs did not stop extraction of metabolites, and relatively high MS signals could be recorded (**Figure S1**).

**Figure 5:**
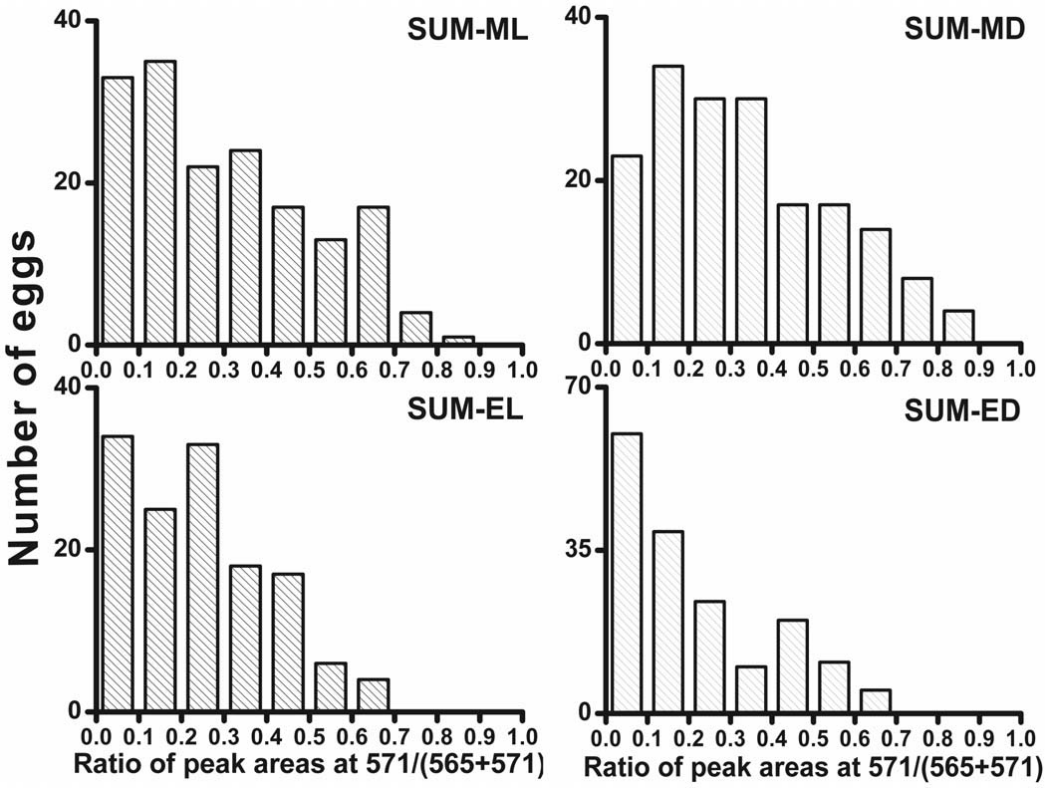
Histograms showing the distributions of labeling levels within the eggs obtained from the fruit flies incubated with ^13^C_6_-glucose (during 12 h) at different illumination conditions and at different times during the day/night cycle. ML – flies incubated during day (starting from the morning) at light; MD – flies incubated during day (starting from the morning) at dark; EL – flies incubated during night (starting from the evening) at light; ED – flies incubated during night (starting from the evening) at dark.

**Table 1:** Notation used in this report.

Abbreviation	Description
ML	flies incubated with ^13^C_6_-glucose during day (9:00 AM–9:00 PM) at light
MD	flies incubated with ^13^C_6_-glucose during day (9:00 AM–9:00 PM) at dark
EL	flies incubated with ^13^C_6_-glucose during night (9:00 PM–9:00 AM) at light
ED	flies incubated with ^13^C_6_-glucose during night (9:00 PM–9:00 AM) at dark

Before the incubations, the flies were entrained to light/dark cycle in the following way: day (light), 9:00 AM–9:00 PM; night (dark), 9:00 PM–9:00 AM.

### Isotopic Labeling of Fruit Flies

When ^13^C_6_-glucose is administered to fruit flies as the only carbon source, the ^13^C atoms are gradually incorporated into cellular metabolites. **Figure 2A** shows the outcome of labeling over 1, 6 and 19 days. We found that – using 1% ^13^C-glucose solution as the only carbon source – uridine diphosphate glucose (UDP-glucose) is promptly labeled with ^13^C, while the labeling of other metabolites – for example, adenosine triphosphate (ATP) – is much slower, and it does not reach completion during several days of incubation. The latter is concluded based on the presence of the non-labeled form of ATP after 19 days (the peak at the *m/z* 506 in **Figure 2A**). Conversely, ^13^C-labeled glucose can readily replace the unlabeled glucose moiety in the molecule of UDP-glucose. Initially, the labeling of UDP-glucose is limited to the glucose (C_6_) moiety, while the UDP moiety remains unlabeled. Biosynthesis of UDP-glucose using UTP and glucose as substrates involves only two reactions, which are catalyzed by two enzymes: hexokinase (EC 2.7.1.1) and UTP-glucose-1-phosphate uridylyltransferase (EC 2.7.7.9; **Figure 2B**) [23]–[25]. This gives the possibility of using partial isotopic labeling of UDP-glucose as an indicator of the early stages of primary metabolism while ^13^C_6_-glucose is the only carbon source available. On the other hand, *de-novo* biosynthesis of adenosine triphosphate (ATP) involves multiple biotransformations, thus only several carbons can be replaced in most ATP molecules during several days of incubation. In order to confirm the incorporation of carbon-13 to the UDP-glucose molecule, we implemented MALDI-MS/MS: As shown in **Figure S4**, the fragment of the unlabeled UDP-glucose (MS *m/z* 565) was found to be unlabeled glucose phosphate (MS/MS *m/z* 241). The tandem MS analysis of the parent ion at the *m/z* 571 (in a sample obtained from a labeled fly) revealed a shift of the fragment peak to the *m/z* 247, which is due to the substitution of 6 ^12^C atoms with ^13^C atoms in the glucose moiety of UDP-glucose. Since the mortality rate was high when the glucose solution was used as the only source of nutrients, we opted for short-term incubations (≤1 day), and – in further experiments – we focused on the measurement of the labeling of glucose moiety in UDP-glucose molecules. It should be noted that UDP-glucose is represented by an MS peak (*m/z* 565 or 571) that does not suffer from spectral/matrix interference.

**Figure 6:**
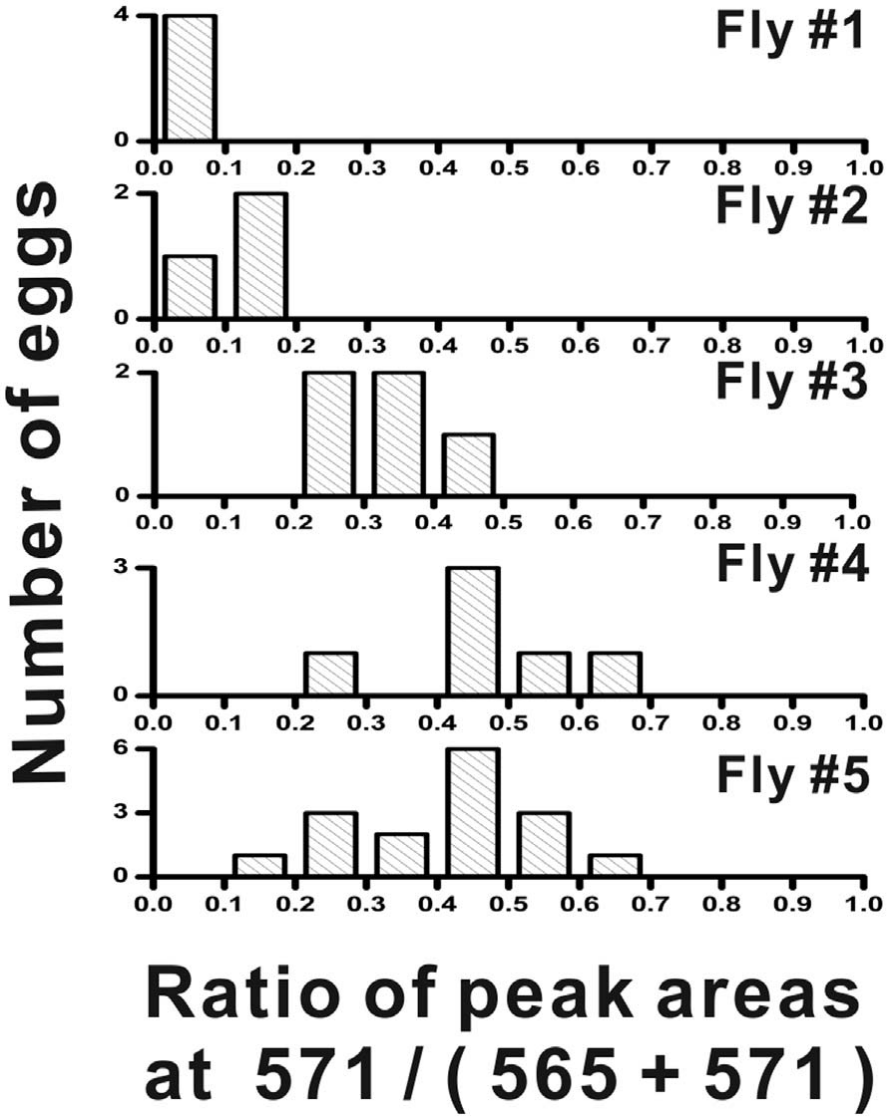
Histograms showing differences in the labeling of eggs among flies from the same treatment. Flies incubated during night (starting from the evening) at dark (ED): arbitrarily selected examples of five single-fly histograms, ordered according to the increasing level of labeling (Fly #1 to #5: top to bottom). All single-fly histograms from this experimental variant are displayed in **Figure S9**.

Using 9-aminoacridine matrix, it is also possible to detect other metabolites (*e.g.* adenosine diphosphate (*m/z* 426), guanosine diphosphate (*m/z* 442), adenosine triphosphate (*m/z* 506), guanosine triphosphate (*m/z* 522), uridine diphosphate glucose (*m/z* 565), uridine diphosphate *N*-acetyl glucosamine (*m/z* 606); **Figure S1**). Selecting UDP-glucose (*m/z* 565) as a target analyte, we further want to show that MALDI-MS is readily applicable to detection of metabolic effects of environmental factors in fruit fly eggs. In future studies, one may also consider studying the labeling patterns of other metabolites by MALDI-MS. However, one can anticipate that the interpretation of results of such studies may not be as straightforward as in the case of UDP-glucose. Another point to consider is that when ^13^C atoms replace ^12^C atoms, the signal-to-noise ratios of the main peaks (corresponding to the unlabeled metabolites) are decreased due to isotopic dilution. Therefore, the experimental strategy described here is most applicable to analysis of metabolites represented by peaks with high signal-to-noise ratios.

### Time Course of UDP-glucose Labeling

Subsequently, the time progress of the incorporation of 6 ^13^C atoms to the glucose moiety of UDP-glucose in fruit fly eggs was studied. Incorporation of ^13^C to UDP-glucose takes approximately 20–30 h: during this time, a gradual increase of the ratio of the labeled UDP-glucose to the total (labeled+unlabeled) UDP-glucose can be observed (**Figure 3**). Based on the sigmoid function fitted to all the data points, the time (*t*_1/2_) after which the labeling of the studied population of eggs was 50% is estimated to be ∼13 h. A considerable variability of the labeling progress can be observed within the population of eggs analyzed at every time point (**Figure 3**). This variability will be discussed later on.

It should also be pointed out that the application of the matrix solution to biological samples, and subsequent analysis in the vacuum compartment of MALDI mass spectrometer may cause some bias to the analytical results. However, this bias is greatly reduced by using isotopic labels: it is unlikely that the enzyme-catalyzed labeling process will proceed with a high rate following the quenching of metabolism with acetonitrile used in the course of the sample preparation.

### The Influence of the Incubation Conditions on the Isotopic Labeling of Eggs

In the following experiment, we investigated the influence of temperature on the labeling of UDP-glucose. Two batches of female fruit flies were incubated under low (21°C) and high (28°C) temperature for 24 h; in both cases, light was on. Following the analysis by MALDI-MS, we found that the labeling level was significantly higher in the flies incubated at 28°C, as compared with the flies incubated at 21°C (**Figure 4**). In another experiment, we also found that illuminating the batch of female flies during the incubation with ^13^C_6_-glucose had strong effect on the labeling of UDP-glucose in eggs: the flies incubated in the dark metabolized much less ^13^C_6_-glucose than the flies incubated under light (**Figure 4**). These observations highlight the feature of the proposed isotope label-aided mass spectrometric method: environmental factors (temperature, light) are reflected in the measured labeling yields of the target metabolite (UDP-glucose). This effect may be due to an alteration of metabolic rates, or change in the feeding habit of flies.

### The Influence of Light/dark Entrainment on the Isotopic Labeling of Eggs

The mechanism of circadian clock has broadly been investigated: a number of previous studies pointed out activation of specific genes which are responsible for the maintenance of circadian rhythm in fruit fly [26], [27]. In the present study, we investigated how the adaptation of fruit fly to light/dark cycle can affect the labeling of UDP-glucose in eggs, irrespective of the treatment (light on/off). It was also interesting to verify the effect of the alteration of culture conditions – from normal (light on during circadian day) to abnormal (light off during circadian day). At the beginning, we entrained the flies with a 24-h light/dark cycle: the photoperiod of 12-h day (9:00 AM –9:00 PM)/12-h night (9:00 PM –9:00 AM). Subsequent incubation with ^13^C_6_-glucose began at the time when the light would be turned on or off: one batch of the flies was incubated under light during 12 h, and the other batch was incubated in the dark during 12 h, irrespective of the light/dark cycle applied during the entrainment period (before the actual experiment). The analysis of single eggs according to the protocol in **Figure 1** followed, and the results are summarized in **Figures 5** **,** and **S5, S6, S7, S8, S9**, and **Tables S1, S2, S3**. **Table 1** summarizes the notation used in this report.

We found that – compared with the flies incubated under normal conditions (light on during day; ML) – the flies incubated under abnormal conditions (light off during day; MD) exhibited similar metabolic activity, *i.e.* in accordance with their biological clock which had been adjusted during the entrainment period. Based on the results of the one-sample Kolmogorov-Smirnov test, we found that none of the data sets in **Figure 5** follows normal distribution. Due to the fact that the labeling is not complete within the 12-h period, corresponding to either circadian day or night (*N.B.* the labeling “half life” at full light: *t*_1/2_ ≈ 13 h), the distributions are asymmetrical and have right-side tails (*Skewness* >0; **Table S1**. Further statistical analysis using the two-sample Kolmogorov-Smirnov test (TSKST) revealed that depriving the fly stock of light during the circadian day (ML *vs.* MD) did not produce a significant change in the distribution of the labeling level of UDP-glucose within a large sample of fly eggs; in other words, the null hypothesis that the ML and MD data sets (**Figure 5**) are from the same continuous distribution was not rejected at the 5% significance level (TSKST *p* = 0.3226; **Table S2**). Another non-parametric test (Wilcoxon rank sum test, WRST) did not reject the null hypothesis that the ML and MD data sets are drawn from identical continuous distributions with equal medians (WRST *p* = 0.1334). In addition, the percentage of eggs with a high labeling level (≥ 0.5) in the samples collected after day-time incubation was comparable in the light- and dark-incubated flies: 21 and 24%, respectively (**Table S3**). Statistical analysis further revealed that the distributions of the data points from the day/light (ML) incubation and the night/light (EL) incubation are different (TSKST *p* = 0.0157; **Table S2**). The data set obtained for the day/dark (MD) incubation also reflects a different distribution than the data set obtained for the night/dark (ED) incubation (TSKST *p*≈0.0000; **Table S2**). The percentage of eggs with a high labeling level (≥ 0.5) was lower in the group of flies incubated with ^13^C_6_-glucose during night (7 and 9%) than during day (21 and 24%), irrespective of the presence of light (**Table S3**). Providing light to the flies during circadian night (EL *vs.* ED) slightly affected the labeling distribution in the studied sample of eggs (TSKST *p* = 0.0172; **Table S2**). This may suggest that the illumination of the fly stock against the entrained circadian rhythm (*i.e.* “light on” during night) has a greater influence on metabolic activity than light deprivation (*i.e.* “light off” during day). Overall, based on the above results, we conclude that the labeling of UDP-glucose in eggs of fruit flies reared under altered illumination follows the internal circadian rhythm, which was learned during the entrainment period preceding the incubation with ^13^C_6_-glucose. It is deduced that the internal circadian rhythm dominantly controls the level of ^13^C incorporated to UDP-glucose despite an acute perturbation to the light/dark cycle.

The differences in the isotopic labeling of eggs raise discussion about the possible source of the dependencies of the labeling rates on the temporal phases within circadian rhythms. In fact, fruit fly exhibits a circadian rest-activity cycle with prolonged intervals of rest, which share many features with mammalian sleep [8], [28]. Previously, the physical activity of fruit flies was studied with a specially designed locomotor assay, which quantifies physical movements of flies [29]. The experimental design of this study has been based on an assumption that the biosynthetic processes observed in unfertilized eggs are linked to the metabolic activity within the bodies of fruit fly from which they have been obtained. The approach implemented in the present work allowed us to obtain information about biosynthesis of one particular metabolite (UDP-glucose). Due to the high labeling rate, observation of the labeling progress during short incubation periods (12 h) was possible (**Figure 3**). The differences in the results obtained following the incubation of flies with ^13^C-glucose at different times, and at different illumination (*e.g.* day/light – ML *vs.* night/dark – ED), may be due to the following reasons: (i) Locomotion [8], gustatory [30] and feeding [11] activities of fruit flies are affected by circadian clock; therefore, ingestion of the isotopically labeled carbohydrate, may be greater during day than at night. (ii) Metabolic activity of individual flies is higher during circadian day than during circadian night, despite the altered light/dark cycle. These alternative interpretations ((i) and (ii)) cannot be dissected using the experimental design implemented in this study. Based on a previous work, the lack of change in the uptake of labeled glucose could be attributed to the lack of change in the feeding activity [11], [30]; however, some contribution of an alteration to the primary metabolism itself should not be excluded.

To the best of our knowledge, the present study is the first one that reveals short-term effects of light/dark entrainment on the biosynthesis of metabolites in metazoan eggs. In future, it would be appealing to develop other microscale assays that would provide complementary data on the molecular composition of individual eggs, and gain further insight to the influence of circadian adaptation on egg metabolism. To this end, one promising experimental approach – complementary to the current one – could be the implementation of microscale nuclear magnetic resonance [31].

### Labeling Variability

In a previous work, stable isotopes were used to study embryonic proteome in fruit fly: pooled samples – composed of multiple embryos – were analyzed [19]. The current study provides information about metabolic activity of individual eggs obtained directly from fly bodies by dissection. While the histograms in **Figure 5** present the data for large numbers of flies (>25) and eggs (>130), it is also interesting to look into the egg-to-egg and/or fly-to-fly variability, which have already become apparent in the labeling time-course study (**Figure 3**). The fly-to-fly variability is illustrated within histograms plotted separately for each fly included in the study (**Figures 6** and **S6, S7, S8, S9**). For example, in **Figure 6** one can easily spot differences between individuals (different histograms), and eggs (multiple bars in the histograms). Although the distribution of labeling in the whole population of fly eggs within one treatment appears flat (for example, **Figure S7**, red bars, for the MD group; *Kurtosis* = 2.26, **Table S1**), the average labeling levels which can be calculated for eggs obtained from every fly are different. These differences are probably the result of phenotypic variability within the population of fruit flies included in this experiment. Close examination of the single-fly histograms in **Figures 6** and **S9**– representing the flies incubated during night at dark (ED) – leads to an observation that although most flies did not incorporate much carbon-13 into their eggs (*e.g.* Flies #1 and #2 in **Figure 6**), there are several outliers – flies with notably higher labeling yield (*e.g.* Flies #4 and #5 in **Figure 6**). While comparable data sets – showing the influence of circadian rhythms on primary metabolism of individual eggs or embryos, and tissue cells – cannot be found in scientific literature, it is worthwhile mentioning that in a study conducted using a mouse model, strong heterogeneity of circadian entrainment kinetics was found not only between different organs, but also within the molecular clockwork of each tissue [32].

It should also be pointed out that eggs from single fly are exposed to the same concentration of ^13^C_6_-glucose; consequently, egg-to-egg variability of the labeling ratios (peak areas at the *m/z* 571/(565+571)) is an intrinsic property of eggs – independent of fly activity and glucose consumption. In each of the single-fly histograms (**Figures S6, S7, S8, S9**, black bars), one can also see the distributions of the labeling ratios within each of the flies studied. The average spreads of the labeling distribution within the population of eggs in every fly included in each treatment were: 0.32±0.15 (s.d.), 0.29±0.11 (s.d.), 0.25±0.10 (s.d.), and 0.26±0.15 (s.d.) in ML, MD, EL, and ED, respectively. Thus, the spreads of the labeling ratios in every fly are typically much smaller than the spread of the labeling ratio calculated for the whole population of eggs included in the analysis: 0.9, 0.9, 0.7, and 0.7 in ML, MD, EL, and ED, respectively (**Figures S6, S7, S8, S9**, red bars); with a tendency towards a higher egg-to-egg variability in the flies incubated with ^13^C_6_-glucose during circadian day.

### Conclusions

The study has shown the feasibility of isotopic labeling of fruit flies with the purpose of pursuing metabolic effects of environmental cues by mass spectrometry. It has demonstrated a link between adaptation to light/dark cycle and biosynthesis of UDP-glucose in eggs of fruit fly. This effect can either be due to the altered feeding habit or metabolic activity; however, further work is needed to verify which of these two factors is more significant. The proposed MALDI-MS and stable isotope-aided protocol was found to produce useful information which reflects the treatment applied to the fly stocks. The main advantage of this experimental approach is that quasi-quantitative data sets can be obtained for single eggs without elaborate sample preparation. Instead of performing absolute quantification of individual metabolites – the ratio of the MS signal corresponding to the labeled metabolite and the sum of the MS signals corresponding to the labeled and the unlabeled metabolite can be used as the proxy for metabolic activity. We believe this method will help to explore other biochemical phenomena related to metabolism in fruit fly as well as other metazoan species (for example, *Caenorhabditis elegans*), which are considered as model organisms in biology.

## Supporting Information

Figure S1 **Wide** ***m/z*** **-range negative-ion MALDI mass spectrum of a single egg obtained from fruit fly.** Metabolites were extracted in situ using 50% acetonitrile solution. MALDI matrix: 9-aminoacridine. Labels of the most prominent peaks: ADP, adenosine diphosphate (*m/z* 426); GDP, guanosine diphosphate (*m/z* 442); ATP, adenosine triphosphate (*m/z* 506); GTP, guanosine triphosphate (*m/z* 522); UDP-glucose, uridine diphosphate glucose (*m/z* 565); UDP-GlcNAc, uridine diphosphate *N*-acetyl glucosamine (*m/z* 606).
(TIF)

Figure S2 **Influence of the concentration of acetonitrile (ACN) in the extraction solution on the signal-to-noise (** ***S/N*** **) ratios.** This experiment included several metabolites (ATP, *m/z* 506; GTP, *m/z* 522; UDP-glucose, *m/z* 565; and a phospholipid, *m/z* 833) extracted from single eggs, and analyzed by MALDI-MS.
(TIF)

Figure S3 **Influence of the concentration of the 9-aminoacridine matrix solution (in acetone) on the signal-to-noise (** ***S/N*** **) ratios of the peaks of various standard compounds.** We found that at 6 mg mL^−1^ 9-aminoacridine (in acetone), the *S/N* value is highest. Therefore, we chose this concentration of 9-aminoacridine for further experiments.
(TIF)

Figure S4 **MALDI-MS and MS/MS analysis of metabolites in the eggs of fruit flies incubated with ^13^C-glucose.** Left: spectra for eggs obtained from flies incubated with ^12^C_6_-glucose. Right: spectra for eggs obtained from flies incubated with ^13^C_6_-glucose for 12 h. Evaluation of the MS/MS data was aided by the METLIN database (Scripps Center for Metabolomics, La Jolla, USA).
(TIF)

Figure S5 **Box plot showing labeling of the eggs in each of the four treatment groups.** ML – flies incubated during day (starting from the morning) at light; MD – flies incubated during day (starting from the morning) at dark; EL – flies incubated during night (starting from the evening) at light; ED – flies incubated during night (starting from the evening) at dark.
(TIF)

Figure S6 **Histograms showing differences in the labeling of eggs among flies from the same treatment (ML – flies incubated during day (starting from the morning) at light; black bars).** Each of the small histograms (black bars) corresponds to a single fruit fly. The cumulative histogram for all the eggs obtained from the ML treatment (red bars) – same as the one displayed in **Figure 5** – is also included for comparison.
(TIF)

Figure S7 **Histograms showing differences in the labeling of eggs among flies from the same treatment (MD – flies incubated during day (starting from the morning) at dark; black bars).** Each of the small histograms (black bars) corresponds to a single fruit fly. The cumulative histogram for all the eggs obtained from the MD treatment (red bars) – same as the one displayed in **Figure 5** – is also included for comparison.
(TIF)

Figure S8 **Histograms showing differences in the labeling of eggs among flies from the same treatment (EL – flies incubated during night (starting from the evening) at light; black bars).** Each of the small histograms (black bars) corresponds to a single fruit fly. The cumulative histogram for all the eggs obtained from the EL treatment (red bars) – same as the one displayed in **Figure 5** – is also included for comparison.
(TIF)

Figure S9 Histograms showing differences in the labeling of eggs among flies from the same treatment (ED – flies incubated during night (starting from the evening) at dark; black bars). Each of the small histograms (black bars) corresponds to a single fruit fly. The cumulative histogram for all the eggs obtained from the ED treatment (red bars) – same as the one displayed in **Figure 5** – is also included for comparison.
(TIF)

Table S1 Statistical data on the samples analyzed in this study. ML – flies incubated during day (starting from the morning) at light; MD – flies incubated during day (starting from the morning) at dark; EL – flies incubated during night (starting from the evening) at light; ED – flies incubated during night (starting from the evening) at dark.
(DOC)

Table S2 Results (*p*-values) of the two-sample Kolmogorov-Smirnov test performed on the data obtained in this study. ML – flies incubated during day (starting from the morning) at light; MD – flies incubated during day (starting from the morning) at dark; EL – flies incubated during night (starting from the evening) at light; ED – flies incubated during night (starting from the evening) at dark. Null hypothesis: the two data sets are from the same continuous distribution. Red color indicates the *p*-values where the null hypothesis is rejected (at the 5% significance level).
(DOC)

Table S3 Numbers of eggs with the labeling level higher or equal to 0.5 in each of the four groups. ML – flies incubated during day (starting from the morning) at light; MD – flies incubated during day (starting from the morning) at dark; EL – flies incubated during night (starting from the evening) at light; ED – flies incubated during night (starting from the evening) at dark.
(DOC)
