# Supplementary figures and images for: Isotope Label-Aided Mass Spectrometry Reveals the Influence of Environmental Factors on Metabolism in Single Eggs of Fruit Fly
Source: PLoS One. 2012 Nov 21;7(11):e50258. doi: 10.1371/journal.pone.0050258 (PMC3503988; doi:10.1371/journal.pone.0050258)

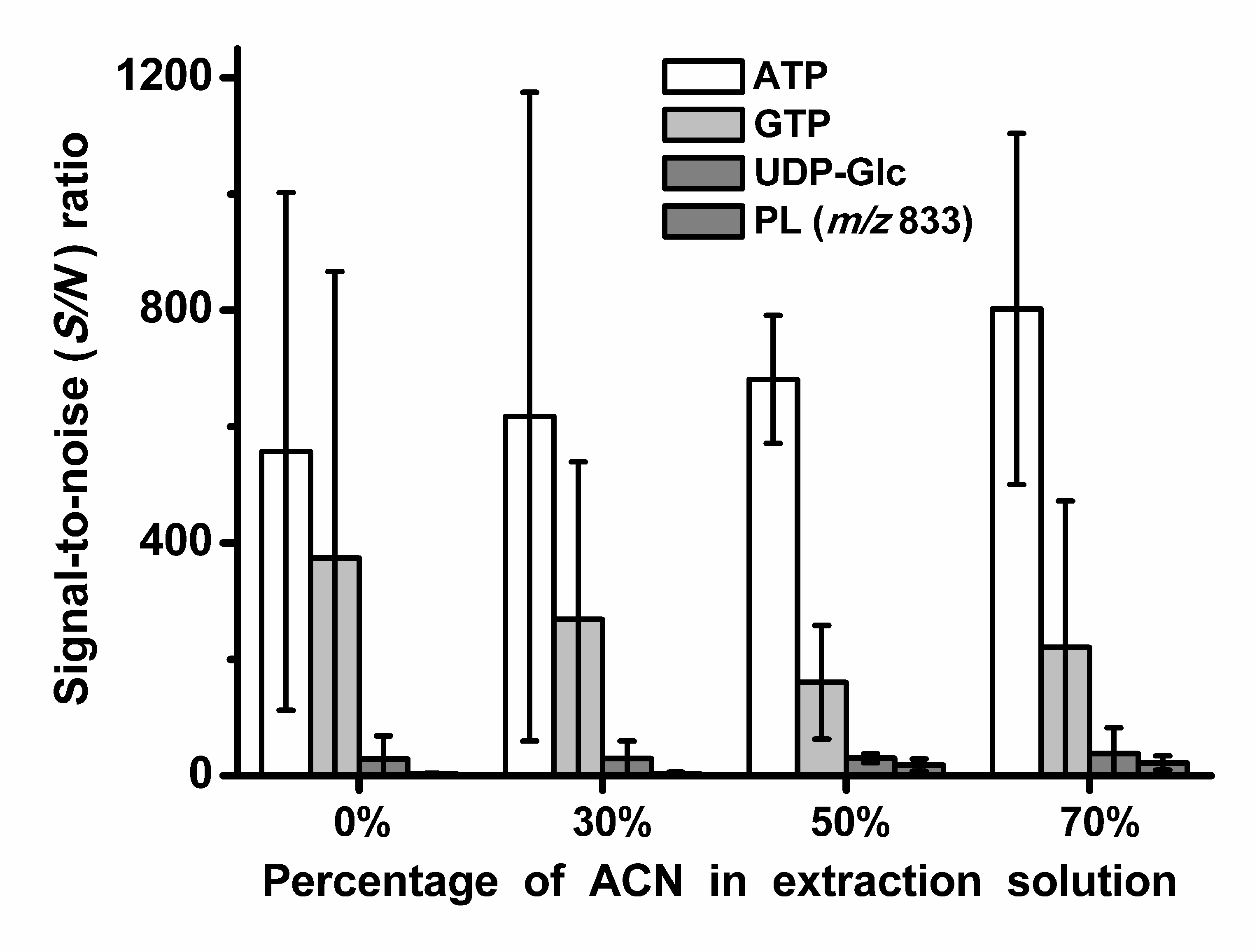

Supplement: Figure S2 — Influence of the concentration of acetonitrile (ACN) in the extraction solution on the signal-to-noise ( S/N ) ratios. This experiment included several metabolites (ATP, m/z 506; GTP, m/z 522; UDP-glucose, m/z 565; and a phospholipid, m/z 833) extracted from single eggs, and analyzed by MALDI-MS. (TIF) [file pone.0050258.s002.tif]

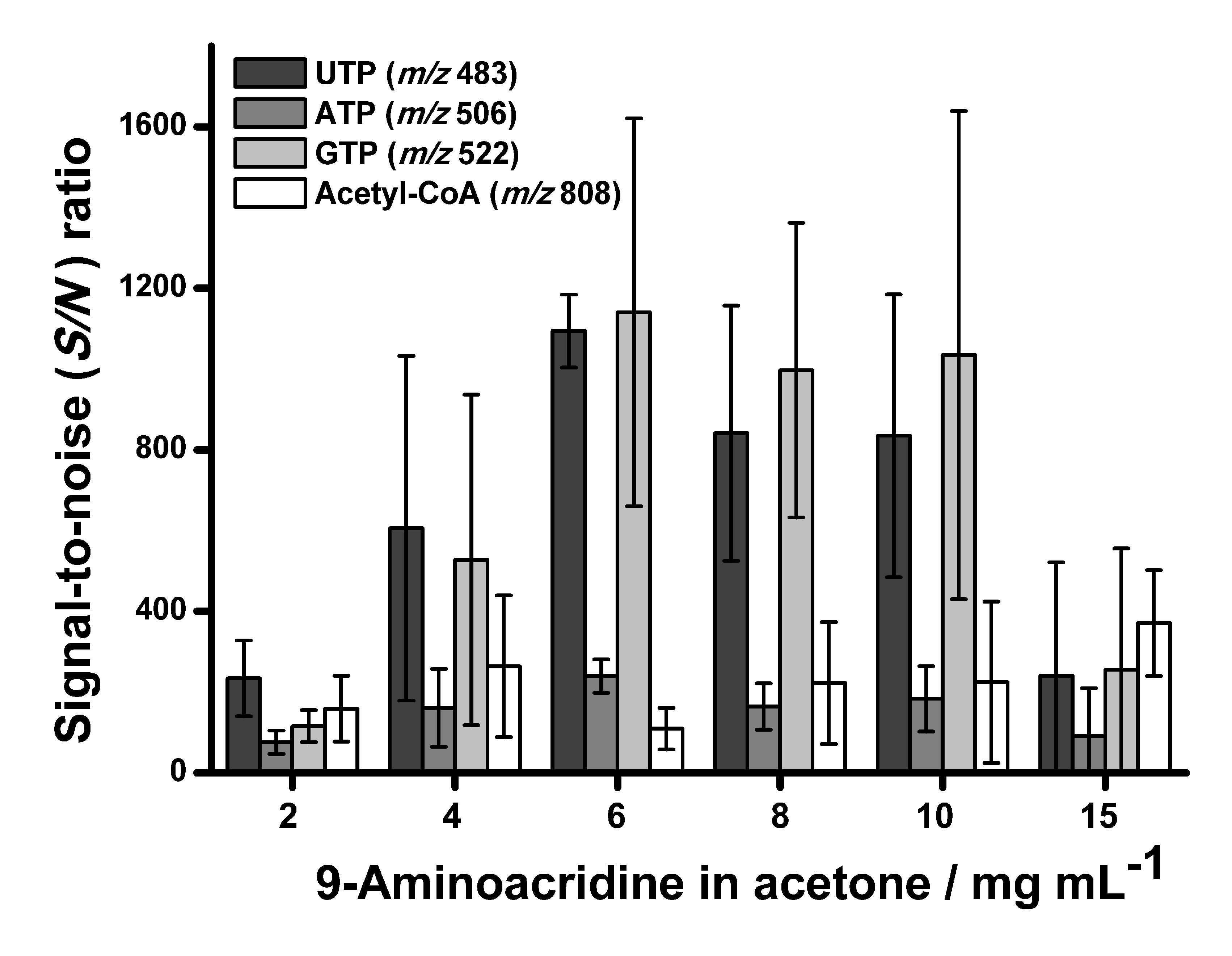

Supplement: Figure S3 — Influence of the concentration of the 9-aminoacridine matrix solution (in acetone) on the signal-to-noise ( S/N ) ratios of the peaks of various standard compounds. We found that at 6 mg mL−1 9-aminoacridine (in acetone), the S/N value is highest. Therefore, we chose this concentration of 9-aminoacridine for further experiments. (TIF) [file pone.0050258.s003.tif]
