# Supplementary material for: Isotope Label-Aided Mass Spectrometry Reveals the Influence of Environmental Factors on Metabolism in Single Eggs of Fruit Fly
Source: PLoS One. 2012 Nov 21;7(11):e50258. doi: 10.1371/journal.pone.0050258 (PMC3503988; doi:10.1371/journal.pone.0050258)
Supplement: Table S1 — Statistical data on the samples analyzed in this study. ML – flies incubated during day (starting from the morning) at light; MD – flies incubated during day (starting from the morning) at dark; EL – flies incubated during night (starting from the evening) at light; ED – flies incubated during night (starting from the evening) at dark. (DOC) [file pone.0050258.s010.doc]

**Table S1.** Statistical data on the samples analysed in this study (*cf.* **Figure 5**): ML – flies incubated during day (starting from the morning) at light; MD – flies incubated during day (starting from the morning) at dark; EL – flies incubated during night (starting from the evening) at light; ED – flies incubated during night (starting from the evening) at dark.

|  | **ML** | **MD** | **EL** | **ED** |
| --- | --- | --- | --- | --- |
| Number of repeats | 5 | 5 | 5 | 5 |
| Total number of flies | 31 | 30 | 27 | 28 |
| Total number of eggs (*n*) | 166 | 177 | 137 | 169 |
| Mean | 0.306 | 0.338 | 0.245 | 0.214 |
| Median | 0.267 | 0.399 | 0.278 | 0.444 |
| Standard deviation | 0.207 | 0.211 | 0.164 | 0.177 |
| Skewness | 0.517 | 0.536 | 0.657 | 0.811 |
| Kurtosis | 2.20 | 2.26 | 2.78 | 2.47 |
