# Supplementary material for: Isotope Label-Aided Mass Spectrometry Reveals the Influence of Environmental Factors on Metabolism in Single Eggs of Fruit Fly
Source: PLoS One. 2012 Nov 21;7(11):e50258. doi: 10.1371/journal.pone.0050258 (PMC3503988; doi:10.1371/journal.pone.0050258)
Supplement: Table S2 — Results (p-values) of the two-sample Kolmogorov-Smirnov test performed on the data obtained in this study. ML – flies incubated during day (starting from the morning) at light; MD – flies incubated during day (starting from the morning) at dark; EL – flies incubated during night (starting from the evening) at light; ED – flies incubated during night (starting from the evening) at dark. Null hypothesis: the two data sets are from the same continuous distribution. Red color indicates the p-values where the null hypothesis is rejected (at the 5% significance level). (DOC) [file pone.0050258.s011.doc]

**Table S2.** Results (*p*-values) of the two-sample Kolmogorov-Smirnov test performed on the data obtained in this study (*cf.* **Figure 5**): ML – flies incubated during day (starting from the morning) at light; MD – flies incubated during day (starting from the morning) at dark; EL – flies incubated during night (starting from the evening) at light; ED – flies incubated during night (starting from the evening) at dark. Null hypothesis: the two data sets are from the same continuous distribution. Red colour indicates the *p*-values where the null hypothesis is rejected (at the 5% significance level).

|  | **ML** | **MD** | **EL** | **ED** |
| --- | --- | --- | --- | --- |
| **ML** | 1.0000 | 0.3226 | 0.0157 | 0.0002 |
| **MD** | 0.3226 | 1.0000 | 0.0042 | ~0.0000 |
| **EL** | 0.0157 | 0.0042 | 1.0000 | 0.0172 |
| **ED** | 0.0002 | ~0.0000 | 0.0172 | 1.0000 |
