# Supplementary material for: Isotope Label-Aided Mass Spectrometry Reveals the Influence of Environmental Factors on Metabolism in Single Eggs of Fruit Fly
Source: PLoS One. 2012 Nov 21;7(11):e50258. doi: 10.1371/journal.pone.0050258 (PMC3503988; doi:10.1371/journal.pone.0050258)
Supplement: Table S3 — Numbers of eggs with the labeling level higher or equal to 0.5 in each of the four groups. ML – flies incubated during day (starting from the morning) at light; MD – flies incubated during day (starting from the morning) at dark; EL – flies incubated during night (starting from the evening) at light; ED – flies incubated during night (starting from the evening) at dark. (DOC) [file pone.0050258.s012.doc]

**Table S3.** Numbers of eggs with the labelling level higher or equal to 0.5 in each of the four groups (*cf.* **Figure 5**): ML – flies incubated during day (starting from the morning) at light; MD – flies incubated during day (starting from the morning) at dark; EL – flies incubated during night (starting from the evening) at light; ED – flies incubated during night (starting from the evening) at dark.

|  | **ML** | **MD** | **EL** | **ED** |
| --- | --- | --- | --- | --- |
| Number of eggs with labelling level ≥0.5 | 35 | 43 | 10 | 16 |
| Share in the total (%; *cf.* **Table S1**) | 21 | 24 | 7 | 9 |
